# Supplementary material for: STARD3: A New Biomarker in HER2-Positive Breast Cancer
Source: Cancers (Basel). 2023 Jan 5;15(2):362. doi: 10.3390/cancers15020362 (PMC9856516; doi:10.3390/cancers15020362)
Supplement: Supplementary file 1 [file cancers-15-00362-s001.zip › Tables S1 and S2.pdf]

Table S1. mRNA expression and DNA copy number for each gene on 51 different breast cancer cell lines. *Data were collected from the Cancer Cell Line Encyclopedia website, Broad Institute of MIT & Harvard.*

Cell line	STARD3 DNA	STARD3 RNA	ERBB2 DNA	ERBB2 RNA	GRB7 DNA	GRB7 RNA	TOP2A DNA	TOP2A RNA
HCC2157_BREAST	0.1564	3.74063845437235	0.1564	5.63829493052793	0.1564	2.96930694015867	-0.3172	5.70830257216067
HS739T_BREAST	-0.0051	3.25807672387796	-0.0051	3.73013280363337	-0.0051	-4.31975230706319	-0.0051	4.3255995827299
HCC38_BREAST	0.7078	4.83359688879628	0.7078	5.9548827461824	0.7078	3.58027605188479	0.7078	7.14424264372184
DU4475_BREAST	0.019	7.1598527096141	0.019	2.18445770149488	0.019	4.02041641170907	0.019	7.2771277426148
MDAMB175VII_BREAST	0.0605	4.37996779284518	0.0605	6.68207346026197	0.0605	3.83357063936939	0.0605	5.59797955459457
YMB1_BREAST	-0.1421		-0.1421		-0.1421		-0.1421	
T47D_BREAST	0.5089	4.72530446715759	0.5089	5.54019935614196	0.5089	3.21078844862327	0.5089	6.95375152798261
HS742T_BREAST	0.0148	3.2110150521835	0.0148	3.13704913657676	0.0148	-5.39582697933276	0.0148	1.82886689518906
KPL1_BREAST	-0.6172	3.68569124041783	-0.6172	4.76386974251808	-0.6172	2.80704030691793	-0.6172	5.60956236391112
MDAMB231_BREAST	0.0894	4.21969512986467	0.0894	3.68157321832868	0.0894	-0.388193728164394	0.0894	7.27015072345395
HCC2218_BREAST	2.7086	7.66630484868248	2.7086	10.3054489294019	2.7086	6.7814142466291	-1.0392	3.87039341247196
HCC1954_BREAST	3.4633	9.8289510304321	3.4633	10.8645630695722	3.4633	9.2044248757925	0.1955	7.08915448210724
CAMA1_BREAST	0.1513	4.38695563351516	0.1513	5.26993721498624	0.1513	2.16993050487664	0.1513	6.56366330194949
HMC18_BREAST	0.4785	4.38825737984208	0.4785	2.80062939135374	0.4785	-3.69812777076163	0.4785	6.7590887625639
AU565_BREAST	2.7059	7.38804094174984	2.7059	10.6940484654529	2.7059	7.5787128929942	1.1804	7.09138873492062
HS578T_BREAST	-0.2712	3.43313713721291	-0.2712	3.18307938383293	-0.2712	-4.73745505381874	-0.2712	5.64634610977208
BT20_BREAST	-0.2918	3.39505867715947	-0.2918	5.11392335112202	-0.2918	2.80711756647533	-0.2918	6.68093528156465
MDAMB361_BREAST	2.2175	7.19202952822555	2.2175	8.61560146354524	2.2175	5.81121993948943	-1.1373	5.46878672132309
MDAMB436_BREAST	-0.555	3.51725548172696	-0.555	2.85581347749338	-0.555	-5.30999595215064	-0.555	5.39903157083463
HCC1143_BREAST	0.1433	4.07595941469333	0.1433	4.11091830262162	0.1433	3.09781289262447	-0.7577	5.5006732949005
HCC1937_BREAST	-0.0018	4.04331520761467	-0.0018	4.63438799130223	-0.0018	3.6424316354395	0.0262	6.91493151789707
HCC1569_BREAST	2.3818	4.97004417169738	3.065	10.0785935405511	3.065	6.37328933572826	-0.1087	6.41174255050665
HCC1395_BREAST	-0.5071	3.93189602393376	-0.5071	2.96718693698403	-0.434	0.282921420263094	-0.434	6.65591477358628
HCC1187_BREAST	-0.3027	3.4654830800928	-0.3027	5.71072100299536	-0.3027	1.99166949086345	-0.3027	6.74667648105317
ZR751_BREAST	-0.0934	3.77062175487836	-0.0934	5.74732125025357	-0.0934	3.37686925989682	-0.0934	6.30642527090342
HCC70_BREAST	-0.5539	3.67819263188211	-0.5539	4.36845410706142	-0.5539	3.17902401647112	-0.5539	6.24890693891603
MCF7_BREAST	-0.9661	3.29188648783331	-0.9661	4.6458804769145	-0.9661	1.35363441226023	-0.3149	5.46644345110357
MCF7_BREAST	-0.9661	3.29188648783331	-0.9661	4.6458804769145	-0.9661	1.35363441226023	-0.3149	5.46644345110357
MDAMB157_BREAST	-0.1929	3.80000100365021	-0.1929	3.42395209518789	-0.1929	-4.0374539635549	-0.1929	7.11015845301091
EFM192A_BREAST	2.7249	8.59930131959605	2.7249	10.7140980178049	2.7249	7.85504172541497	0.8409	7.46975545230188
EVSAT_BREAST	0.1365		0.1365		0.1365		0.6773	
MDAMB453_BREAST	1.1586	5.56520994911134	1.1586	7.32570072243021	1.1586	4.54636968309572	-1.0613	5.33303384278408
EFM19_BREAST	0.6518	5.2169477048811	0.6518	6.54989580461561	0.6518	3.47788101968784	0.6518	7.25859493005535
BT474_BREAST	2.6813	7.87067955753244	3.956	9.7643424545196	2.8149	7.09369528404834	-0.2824	6.03378777919526

HS606T_BREAST	-0.0002	3.55221468031118	-0.0002	3.78199487134591	-0.0002	-4.67899829465597	-0.0002	4.57808920550719
MDAMB415_BREAST	-0.0879	4.18439882854458	-0.0879	5.05475880978133	-0.0879	3.2657426528061	-0.0879	4.63988792675961
MDAMB468_BREAST	-0.3901	4.02676268270358	1.6605	3.57698652531407	-0.358	2.93823121592141	-0.358	6.1436466545837
BT549_BREAST	-0.4759	3.42163431011318	-1.351	3.1576655024163	-0.3831	-1.86834223664296	-0.3831	6.51711956957627
CAL851_BREAST	0.1725	4.23951418615034	0.1725	4.69243001536711	0.1725	3.38726770217246	0.1725	5.75039265194264
HMEL_BREAST	0.0144	4.49089885037763	0.0144	4.405345092458	0.0144	2.91820167160757	0.0144	5.40640770612766
HCC1806_BREAST	-0.1256	3.56085034219227	-0.1256	4.42364965757414	-0.1256	3.48372329183038	-0.1256	5.76913363430976
UACC812_BREAST	2.3893	7.89966927731364	4.3523	10.3662170779417	2.3946	7.89569638197368	1.5054	7.26917620038326
UACC893_BREAST	3.0726	8.72958050107719	3.0726	10.8356523179996	3.0726	8.69945701862981	-0.4519	4.97521395428991
HDQP1_BREAST	0.0894	4.02531650672027	0.0894	4.82636402524222	0.0894	3.41077700869197	0.0894	5.64156813530351
HCC1419_BREAST	2.5161	8.60776205788882	2.5161	10.7187986369465	2.5161	8.14951797197079	-0.7071	5.5550496623068
HS274T_BREAST	0.0059	3.48620407761891	0.0059	3.92191301338029	0.0059	-4.40964001538879	0.0059	6.59511654084131
ZR7530_BREAST	2.7367	8.25017001655364	2.7367	10.5732000246994	2.7367	7.75750446121823	-0.8426	5.17238004661442
HS343T_BREAST	0.0135	3.42626257891994	0.0135	3.5280036895078	0.0135	-4.68550249706072	0.0135	5.92231653786233
CAL51_BREAST	0.0298	3.76819422084561	0.0298	5.24723246371957	0.0298	3.14499906610743	0.0298	7.0210275675462
CAL120_BREAST	-0.1496	3.53044420208868	-0.1496	3.56941005015947	-0.1496	-0.338644954942298	-0.1496	5.81415762664775
JI MT1 _BREAST	2.4607	7.14801194873919	2.4607	7.60529616186604	2.4607	6.45840260788093	-0.317	6.1381380314502

Table S2. Clinical and pathological characteristics according to STARD3 expression.

	STARD3- (N=15)	STARD3+ (N=97)	Total (N=112)	P-value
Initial T stage (TNM)				
T1	0 (0.0%)	20 (20.6%)	20 (17.9%)	0.093
T2	13 (86.7%)	62 (63.9%)	75 (67.0%)	
T3	0 (0.0%)	9 (9.3%)	9 (8.0%)	
T4	2 (13.3%)	6 (6.2%)	8 (7.1%)	
Initial N stage (TNM)				
N0	7 (46.7%)	44 (45.4%)	51 (45.5%)	0.991
N1	7 (46.7%)	47 (48.5%)	54 (48.2%)	
N2	1 (6.7%)	6 (6.2%)	7 (6.2%)	
Tumoral grade				
SBR I-II	9 (60.0%)	35 (36.1%)	44 (39.3%)	0.078
SBR III	6 (40.0%)	62 (63.9%)	68 (60.7%)	
ER H-score				
Mean (SD)	173.067 (128.326)	129.428 (123.067)	135.272 (124.091)	0.206
Range	0 - 300	0 - 300	0 - 300	
PR H-score				
Mean (SD)	54.567 (102.943)	64.232 (102.043)	62.938 (101.751)	0.734
Range	0 - 300	0 - 300	0 - 300	
Ki67 expression				
Unknown	2	1	3	0.223
< 30%	5 (38.5%)	22 (22.9%)	27 (24.8%)	
≥ 30%	8 (61.5%)	74 (77.1%)	82 (75.2%)	
Histological subtype				
Ductal	13 (86.7%)	94 (96.9%)	107 (95.5%)	0.175
Lobular	1 (6.7%)	2 (2.1%)	3 (2.7%)	
Lymphoïd stroma	1 (6.7%)	1 (1.0%)	2 (1.8%)	
Associated ductal carcinoma in situ				
No	9 (60.0%)	48 (49.5%)	57 (50.9%)	0.448
Yes	6 (40.0%)	49 (50.5%)	55 (49.1%)	

Legend: STARD3 = StAR-related lipid transfer domain-3; SD = standard deviation; ER = estrogen receptors; PR = progesterone receptors; SBR = Scarff-Bloom-Richardson
