# Supplementary figures and images for: STARD3: A New Biomarker in HER2-Positive Breast Cancer
Source: Cancers (Basel). 2023 Jan 5;15(2):362. doi: 10.3390/cancers15020362 (PMC9856516; doi:10.3390/cancers15020362)

Supp S1 anti-STAR3

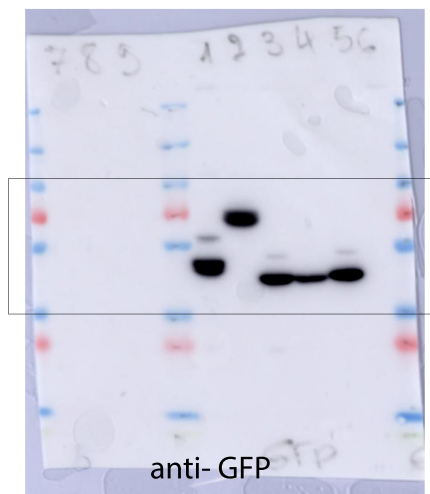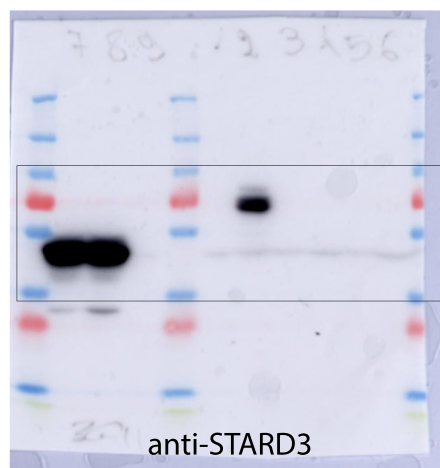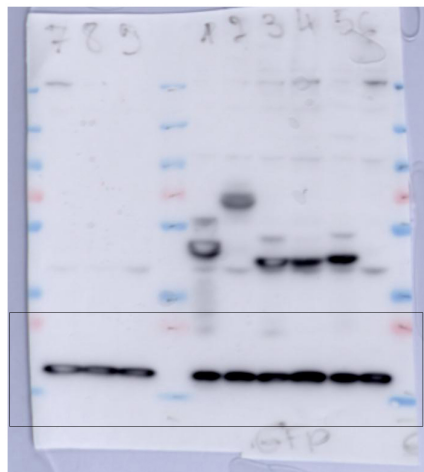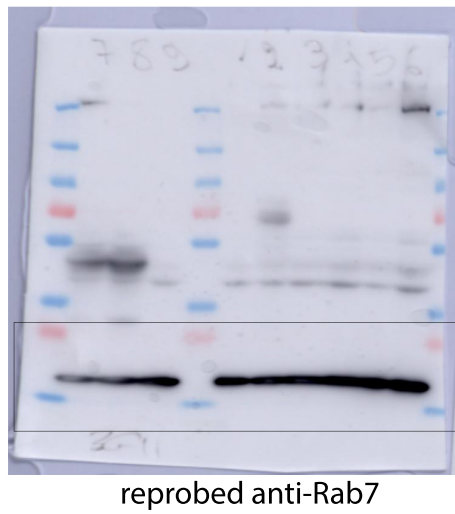

Supplement: Supplementary file 1 [file cancers-15-00362-s001.zip › Figure S1. Whole western blot figures.pdf]
